# Five new species of *Triotemnus* (Coleoptera, Curculionidae, Scolytinae) from Morocco and Yemen

**DOI:** 10.3897/zookeys.56.526

**Published:** 2010-09-17

**Authors:** Miloš Knížek

**Affiliations:** Department of Forest Protection, Forestry and Game Management Research Institute, Jíloviště - Strnady, CZ-156 04 Praha 5 - Zbraslav, Czech Republic

**Keywords:** Bark beetles, Dryocoetini, Palaearctic region, Morocco, Yemen, Socotra

## Abstract

Fivenew species of the genus Triotemnus from Morocco and Yemen are described. Triotemnus is a new genus of Scolytinae for the Yemen region. External morphology of the new species and all morphologically related species of the genus were studied. While the new species from Morocco are morphologically similar to the known species from the corresponding region, all three newly described species from Yemen, mainly two of them living in Socotra, are morphologically very different from all other known species of the genus. Geographical distribution and the probability of endemicity are discussed.

## Introduction

The genus Triotemnus Wollaston, 1864, comprises 11 species distributed in the Palaearctic, Afrotropical and Oriental regions (Wood and Bright 1992, Bright and Skidmore 1997, 2002; Gatti and Pennacchio 2004). In total, 7 species are known from the Palaearctic region. From these, only two species, Triotemnus subretusus Wollaston, 1864 and Triotemnus ulianai Gatti & Pennacchio, 2004, occur in Europe, and five species are known from North Africa – Triotemnus antoinei Peyerimhoff, 1949, Triotemnus lepineyi Balachowsky, 1949 and Triotemnus longicollis Peyerimhoff, 1925 from Morocco, Triotemnus grangeri Peyerimhoff, 1919 from Algeria and Triotemnus subretusus Wollaston, 1864 from the Canary Islands. Only one species is known from Palaearctic part of India – Triotemnus scrofa Schedl, 1975, from Uttar Pradesh. This species, together with Triotemnus pilicornis Wood, 1992, occur in Oriental region (India, and Triotemnus scrofa also in Sri Lanka). Other species of the genus – Triotemnus aethiopicus Eggers, 1936, Triotemnus striatus Eggers, 1936 and Triotemnus villiersi Schedl, 1958 live in Afrotropical region. Interestingly, all the species were described in separate publications (Balachowsky 1949b; Eggers 1936a; Eggers 1936b; Gatti and Pennacchio 2004; Peyerimhoff 1919; Peyerimhoff 1925; Peyerimhoff 1949; Schedl 1958; Schedl 1975; Wollaston 1864 and Wood 1992). That fact, as well as the rather scarce literature on species and genus level, reflects the relative rareness of all the species. Some species are also rare in museum collections and are very seldom collected by entomologists. Collecting of these species in the field is apparently difficult, but many specimens can nevertheless be obtained by raring when infested parts of the host plants are collected.

Even though the fauna and flora of Socotra shows high endemicity, there are in some cases affinities to the Ethiopian and Indian fauna, perhaps due to a common geological history of these areas. Socotran species of Triotemnus differ distinctly from all other species of the genus. Including the information about their biology, these species are suggested as endemic and the possibility for occurring elsewhere is very unlikely. On the contrary, the new species from Morocco are morphologically very similar to known species from that region. Because of the morphological uniformity, one may suggest that species from this region diversified rather recently, and have had little time for morphological differentiation (see e.g. Jordal and Hewitt 2004; Jordal et al. 2004, for similar examples). Even though the new species were always collected from just one location, the broader distribution of their host plants makes occurrence in other areas likely (all known species of Triotemnus are living in “small” shrubs, like Euphorbia spp. and Bupleurum spp.). On the other hand makes the geographical conditions, such as deep valleys and the meteorological conditions of high mountains, relatively suitable for long term separation of local populations.

## Methods

Newly discovered species were compared to all known species in the genus, except Triotemnus ulianai Gatti & Pennacchio, 2004, which was not available for the study. The significance of morphological differences was affirmed by examining long series of specimens. Descriptions use the terminology from e.g. Wood 1986, Wood 1992 and/or Jordal 2009. Specimens were collected from their host plants directly in the field, and by rearing in the lab. When possible, the gallery system was also studied by dissecting host plant tissue. Internal characters of the beetles were not studied.

## Descriptions of the new species

### Triotemnus

Wollaston

Cladoctoporcus  Schedl, 1975. Type species Cladoctoporcus scrofa Schedl, 1975

#### Type species.

Triotemnus subretusus Wollaston, 1864

#### Note.

The position of the genus Triotemnus Wollaston within the higher systematics of bark beetles has changed frequently since its origin. The very first attempt to range the genus within the higher systematics was made by Ferrari 1867, who recognized 6 groups of scolytids and Triotemnus was placed into Group 5 – Tomicides. Later, many different authors placed the Triotemnus in different tribes, but three main transfers were particularly important. Hagedorn 1908 suggested that the genus belongs to the family Crypturgidae, followed by Hopkins 1915, which ranked it at subfamily level. This was followed by Schedl 1932, which placed it into the tribe Crypturgini LeConte, 1876. Later it was transferred to the tribe Thamnurgini Nüsslin, 1911 by Balachowsky 1949a and afterwards into Dryocoetini Lindemann, 1876 by Wood 1986, in which it is remaining in the most modern system and the most important recent papers until now, with one exception of Pfeffer 1995, which followed Balachowsky 1949a. There is only one synonymy at the generic level, the monobasic genus Cladoctoporcus Schedl, 1975 was synonymized by Wood (1984). All other species currently included in the genus were originally described in Triotemnus, except Triotemnus grangeri (Peyerimhoff, 1919), which was originally described under the genus Lymantor Løvendal, 1889 and was transferred to Triotemnus by Peyerimhoff in 1949.

#### Diagnosis.

Length 1.2 – 2.3 mm, slender, cylindrical body form, reddish to dark brown, antennae and legs lighter. Male frons flattened to concave. Eyes emarginate on anterior margin around the antennal insertion. Each mandible with short or long pointed tooth-like process directed upward. Antennal funicle 4- or 5-segmented (3-segmented in Triotemnus scrofa), antennal club longitudinally oval, usually with two sutures in apical part on anterior side. Pronotum oval or cylindrical, longer than wide, feebly declivous anteriorly, distinctly punctured, usually with impunctate median longitudinal area, not armed on basal margin. Scutellum visible, rather small, flush with elytra. Elytra cylindrical, sometimes widened or narrowed posteriorly, more or less deeply punctured in striae, finely punctured in interstriae, interstriae flat, usually smooth or minutely granulate, elytral declivity regularly rounded or flattened, sometimes with distinct lateral edges. Vestiture usually of long erected hair-like setae, these setae may be longer anteriorly, laterally and posteriorly. Procoxae contiguous, lateral margin of protibia armed by 4–5 socketed teeth. Female similar to male, except frons slightly convex, mandibles without the tooth-like processes.

Triotemnus Wollaston, 1864 differs from closely related genera (e.g. Thamnurgus Eichhoff, 1864, Xylocleptes Ferrari, 1867 and Lymantor Løvendal, 1889) mainly by modified frons in males with tooth-like processes on mandibles, feebly if at all declivous pronotum, which is unarmed on anterior margin. Tiarophorus Schreiner, 1882 has much longer pronotum and strongly, but differently modified frons in males and 6-segmented antennal funicle. Some species ranged in Tiarophorus (Wood and Bright 1992) are recently considered under Pseudothamnurgus Eggers, 1912 (e.g. Pfeffer 1995). Solving the relationship of this genus/species with Triotemnus remains for the future study. Taphronurgus Reitter, 1913 has no tooth-like processes on mandibles in males. The most similar Cynanchophagus Aksent’ev, 1987 has much longer pronotum and 7–9 socketed protibial teeth (Mandelshtam et al. 2006).

#### Triotemnus pseudolepineyi

Knížek sp. n.

urn:lsid:zoobank.org:act:B48319BF-8FAA-4597-A179-4410273EBFC1

Figs 1, 2 

##### Type material.

Holotype male, pinned, with labels as follows: “Morocco, 31. 5. 1999/ High Atlas, Toubkal massif/ Chamharouch/ Jan Batelka lgt.“ 31°5'35N; 7°54'22W. Allotype female, pinned: the same data as the holotype. Paratypes: 125 males, 113 females: the same data as the holotype.

Holotype and Allotype deposited in the collection of National Museum in Prague, 190 paratypes in the author’s collection, 4 paratypes in Naturhistorisches Museum, Wien, 4 paratypes in Muséum d’Histoire Naturelle, Genève, 40 paratypes in coll. J. Batelka (Praha).

##### Diagnosis.

This species is morphologically distinct from all other species of the genus due to the strongly developed callous-like lateral edges of elytral declivity. It is the most closely related to Triotemnus lepineyi Balachowsky, from which it differs also by the more elevated lateral edges on elytral declivity, which are clearly higher than slightly elevated suture and by which the elytra appear deeply bisulcate. Elytra are also more narrowly rounded on posterior quarter in Triotemnus pseudolepineyi. The elevated callous-like longitudinal process on the lateral margin terminated before reaching the posterior margin of elytra from lateral view.

**Figures 1–12.**
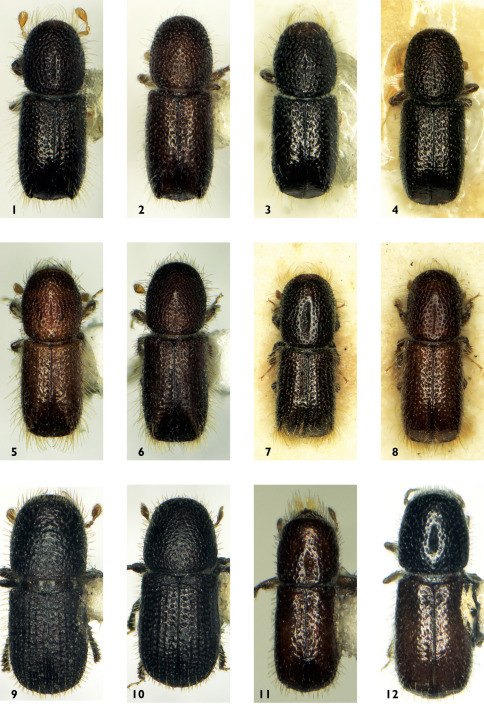
Dorsal view. **1** Triotemnus pseudolepinei – male **2** Triotemnus pseudolepinei – female **3** Triotemnus lepinei – male **4** Triotemnus lepinei – female **5** Triotemnus batelkai – male **6** Triotemnus batelkai – female **7** Triotemnus grangeri – male **8** Triotemnus grangeri – female **9** Triotemnus socotraensis – male **10** Triotemnus socotraensis – female **11** Triotemnus kabateki – male **12** Triotemnus yemenensis – female.

##### Description.

###### Male.

**Length** 1.30–1.80 mm (1.72 mm in holotype), 2.50–2.69 times longer than wide (2.61 in holotype). Colour brown to dark brown if fully coloured. **Head**. Frons concave, excavated, concavity may differ from deep excavation up the level just above upper edge of the eyes, which is smooth, strongly shining, very finely and sparsely punctured, uniformly shagreened, to rather shallow, densely and rather deeply punctate, excavation divided from vertex by semi-carinate costa, this elevated in the middle forming wide-based tubercle-like process; vertex shining, deeply and rather densely punctured; vestiture of very long sparse erected gold hair-like setae situated on the lateral edges of the frontal excavation mainly. Eyes emarginate on anterior margin around the antennal insertion, rather small. Each mandible with short, strong and pointed tooth-like process directed upward, reaching just the lower level of frontal excavation. Antennae light brown, antennal funicle 4-segmented, antennal club longitudinally oval, anterior side with one transverse suture just above the middle, second suture slightly marked in apical half by row of setae, basal half corneous, posterior side without sutures. **Pronotum** 1.08–1.15 times longer than wide (1.12 in holotype), sub-oval, widest in the middle of its length, weekly declivous in front, summit not clearly marked, punctato-granulate on anterior declivity, frontal edge rounded, basal angles broadly rounded, posterior margin rounded, disk shining, strongly and rather densely punctate on disk and lateral areas, interspaces very finely shagreened, median longitudinal area impunctate; vestiture of sparse hair-like setae, becoming very long anteriorly and posteriorly. **Elytra** 1.43–1.51 times longer than wide (1.50 in holotype), 1.42–1.56 times longer than pronotum (1.42 in holotype), sub-parallel on basal three-fourths, widest in posterior part where are slightly wider then pronotum, then converging to rounded apex; basal edge rounded, not armed; scutellum visible, very small, smooth and shining, flush with elytra; first striae slightly impressed on the whole length of the elytral disc, other striae not impressed; elytral striae deeply punctured, interspaces slightly larger then the diameter of the punctures; interstriae less densely and less deeply punctured, shining. Elytral declivity regularly rounded and elevated at suture, bisulcate, lateral edges strongly elevated, fourth interstriae developed into a large callous-like longitudinal and slightly undulated process, elevation not reaching the posterior margin, lateral elevated edges much higher than slightly elevated suture, interspace between the suture and elevated lateral edges depressed, smooth, shining, very finely and sparsely punctuate at the striae, and impunctate on interstriae. Elytral vestiture consisting of sparse hair-like semi-erected setae on disk, each about 1.5 times longer than the distance between strial punctures, much longer (approximately twice) hair-like setae on lateral and posterior margins; sulcate part of the elytral declivity without setae except short oblique setae along the suture (often abraded). **Legs brown**. Procoxae subcontiguous, prosternal process very narrow, long and sharply pointed; mesocoxae separated by the width of scapus, mesoventral process descending, narrow and bluntly pointed; metacoxae separated slightly more than mesocoxae. Pro-, meso- and metatibiae on outer lateral margin usually with 6 socketed teeth, mesotibiae sometimes with 5 socketed teeth, variable between specimens within series.

###### Female.

**Length** 1.26–1.85 mm (1.56 mm in allotype), 2.58–2.70 times longer than wide (2.63 in allotype). **Head** similar to male, but mandibulae not armed by tooth-like processes, frons convex, slightly flattened just above the epistoma, dull, very densely punctato-ganulate, becoming less pronounced in the flattened area, on vertex with a wide tubercle-like process; vestiture of very fine, short, erect yellowish hair-like setae directed forward, becoming denser toward epistomal margin. Antennal club different from male, short and oval. **Elytra** withlateral parts of declivity less strongly developed, but higher then elytral suture.

##### Etymology.

Name of this new species is derived from the morphologically nearest species named and described as “lepineyi” by Balachowsky (1949b) and using of the Greek term “pseudos” – pretended.

##### Biology.

Specimens were collected from wilting shrubs of Bupleurum spinosum (Apiaceae). The species is phloeophagous. Maternal galleries were not clearly visible due the full consumption of phloem and sapwood by the larvae. Before pupation the larvae bore into the sapwood forming up to one centimetre long internal pupation galleries. Larval galleries were generally filled with yellow frass in their whole length.

##### Distribution.

Morocco (High Atlas), endemic. Even though the species is suggested as endemic here, it is possible that it may be found in other areas, mainly within Morocco and Algeria, because the host plant is found in neighbouring areas.

#### Triotemnus batelkai

Knížek sp. n.

urn:lsid:zoobank.org:act:3A52C4BA-3D76-4ED1-A881-97CDEE161230

Figs 5, 6 

##### Type material.

Holotype male, pinned, with labels as follows: “Morocco, 2260–2350m/ Tizi-n-Tichka pass, 30.VI-1.VII.1998, ex larva VIII. 1999/ J. Batelka & H. Batelková lgt.“ 31°24'58N; 7°23'34W. Allotype female, pinned: the same data as the holotype. Paratypes: 23 males, 63 females: the same data as the holotype.

Holotype and Allotype deposited in the collection of National Museum in Prague, 62 paratypes in the author’s collection, 2 paratypes in Naturhistorisches Museum, Wien, 2 paratypes in Muséum d’Histoire Naturelle, Genève, 20 paratypes in coll. J. Batelka (Praha).

##### Diagnosis.

The species is morphologically distinct from the most closely related species Triotemnus grangeri (Peyerimhoff), which in Triotemnus grangeri has the lateral edges of elytral declivity more strongly developed, the declivital flattened area is much wider, the vestiture on frontal edge of pronotum and lateral and posterior margins of elytra is slightly longer, and the uniseriate setae along the suture on the elytral declivity are much more stout and longer.

##### Description.

###### Male.

**Length** 1.43–2.00 mm (1.63 mm in holotype), 2.55–2.86 times longer than wide (2.76 in holotype). Ferruginous to dark brown if fully coloured. **Head**. Frons shallowly concave up to the level just above upper edge of the eyes, concavity rather deeply punctured except very finely punctured, nearly impunctate area above epistoma, shining, very finely shagreened, excavation divided from vertex by rounded costa elevated in the middle forming a short transversely rounded keel; vertex semi-shining, deeply and rather densely punctured; vestiture of very long, erected, golden hair-like setae situated on the lateral edges of the frontal excavation mainly, these on concavity of about half length. Eyes emarginate on anterior margin around the antennal insertion, rather small. Each mandible with a short and strong (wide based) pointed tooth-like process directed upward, reaching just the lower level of frontal excavation. Antennae yellowish, antennal funicle 4-segmented, antennal club longitudinally oval, anterior side with one transverse suture just above the middle, apical half covered by dense setae, basal half corneous, posterior side without sutures. **Pronotum** 1.14–1.21 times longer than wide (1.20 in holotype), sub-oval, widest in the middle of its length, frontal edge rounded, basal angles broadly rounded, posterior margin rounded; anterior disk weekly declivous, summit not clearly marked, approximately in the frontal third, disk shining, strongly and densely punctate along an impunctate and smooth median longitudinal area, punctato-granulate on the remaining disk, granulation becoming stronger laterally and frontally mainly, interspaces finely rugose; vestiture of sparse semi-erect hair-like setae directed towards impunctate medial line, setae becoming much longer anteriorly. **Elytra** 1.39–1.64 times longer than wide (1.51 in holotype), 1.32–1.35 times longer than pronotum (1.33 in holotype), sub-parallel on basal three-fourths, widest in posterior half, than converging to broadly rounded (sub-straight) apical margin; base of elytrae rounded, not armed; scutellum visible, but very small, smooth and shining, flush with elytra; elytral striae rather deeply sparsely and regularly punctured, not impressed, interspaces slightly larger then the diameter of the punctures; interstriae less densely and less deeply punctured; elytral suture slightly elevated on nearly its entire length except on the basal fifth of elytra. Elytral declivity regularly rounded at suture, otherwise flattened up to the clearly defined lateral edges which are armed by a narrow and regularly elevated ridge, the costate ridge slightly undulated by a small blunt tubercles, lateral elevated edges slightly higher than slightly elevated suture, declivital disc smooth, shining, impunctate except distinctly punctuate in continuation of striae 1 and 2; elytral apex broadly round (nearly straight from dorsal view), armed by a slightly elevated costa. Elytral vestiture of two types, sparse and very long (more than two times longer than distance between the strial punctres) hair-like erected setae and semi-erected half long hair-like setae, setae becoming much longer (approximately double length) and dense laterally and posteriorly and on lateral margins of the declivity; disk of the elytral declivity without setae except sparse uniseriate short stout oblique setae along the suture, these directed toward the declivital margins (these setae often abraded). **Legs** light brown. Procoxae contiguous, prosternal process narrow, sharply pointed; mesocoxae nearowly separated by distance less then width of scapus, mesoventral process descending, narrow and bluntly pointed; metacoxae separated by twice the width between mesocoxae. Number of tibial socketed teeth on outer lateral margin varying between the specimens: protibiae with 5–7 socketed teeth, mostly 6, meso- and metatibiae with 6 socketed teeth.

###### Female.

**Length** 1.43–1.92 mm (1.70 mm in allotype), 2.60–2.87 times longer than wide (2.62 in allotype). **Head** similar to male, but mandibulae not armed by tooth like processes, frons convex, slightly flattened just above the epistoma, semi-matt, very densely punctato-ganulate, this sculpture becoming more fine on the flattened area, vestiture of golden, rather dense, long, erected hair-like setae becoming gradually longer towards the epistomal margin. Antennal club oval, but shorter than in male. **Elytra** with lateral margins of the elytral declivity slightly less sharply developed.

##### Etymology.

The new species is dedicated to my colleague Jan Batelka, who collected the specimens. Jan Batelka is the leading scientist in taxonomy of the beetle family Rhipiphoridae.

##### Biology.

Specimens were collected from wilting shrubs of Bupleurum spinosumр additional specimens were obtained by rearing in the lab. The newly described species is phloeophagous. The gallery system was not studied.

##### Distribution.

Morocco (High Atlas), endemic. Even though the species is suggested as endemic here, it is possible that it may be found in other areas, mainly within Morocco and Algeria, because of the host plant occurrence in neighbouring areas.

#### Triotemnus socotraensis

Knížek sp. n.

urn:lsid:zoobank.org:act:76EB4C08-A10F-43BF-A279-AEA3C652C85F

Figs 9, 10 13 

##### Type material.

Holotype male, pinned, with labels as follows: “Yemen, Soqotra Is., 10 km W HADIBOH/ 23.xi.-11.xii.2003, 10–70 m [GPS]/ leg. P. Kabátek, ex larve“, 12°39’N; 53°57’E; “YEMEN-SOQOTRA/ 2003; Expedition: Jan Farkač, Petr Kabátek & David Král“; “Host plant: Euphorbia arbuscula”. Allotype female, pinned: the same data as the holotype. Paratypes: 69 males, 70 females: the same data as the holotype; 7 males, 5 females: “Yemen, Soqotra Is., SUQ E/ env. sand dune, 22.XI.2003, 12°40'02N; 54°03'45E, 20–170 m [GPS]/ leg. P. Kabátek, ex larve“; “YEMEN-SOQOTRA/ 2003; Expedition: Jan Farkač, Petr Kabátek & David Král“; “Host plant: Acacia pennivenia”.

Holotype and Allotype deposited in the collection of National Museum in Prague, 147 paratypes in the author’s collection, 2 paratypes in Naturhistorisches Museum, Wien, 2 paratypes in Muséum d’Histoire Naturelle, Genève.

**Figures 13–14.**
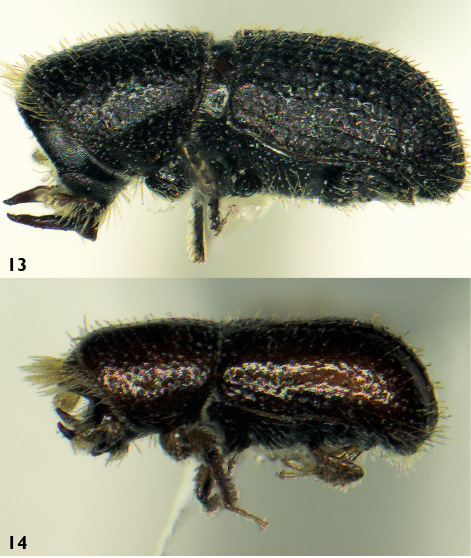
Lateral view. **13** Triotemnus socotraensis – male **14** Triotemnus kabateki – male

##### Diagnosis.

Triotemnus socotraensis is morphologically very distinct from all other species of the genus due to the very stout body and morphology of elytra. There is no other known species with such strongly punctate elytral striae and granulato-punctate interstriae. It is also unique by the uniformly dark, nearly black, colour.

##### Description.

###### Male.

**Length** 1.41–1.89 mm (1.76 mm in holotype), 2.23–3.28 times longer than wide (2.38 in holotype), short and cylindrical, very stout, colour dark brown to black. **Head**. Frons broadly concave up to vertex, slightly flattened transversely above the epistoma, matt, concavity shallowly, but very distinctly and rather densely punctured, whole surface including inside and between punctures strongly shagreened, excavation separated from vertex by a well defined transverse costal ridge, which is somehow undulated due minute blunt tubercles, vertex shagreened; vestiture of sparse, short, semi-erect, golden hair-like setae, these becoming more dense and longer around the insertion of antennae. Eyes rather deeply emarginate on anterior margin, their fronto-dorsal margin slightly protuberant above the costal ridge of excavation of frons. Each mandible with a very long sabre-shaped process directed anteriorly and curved inward, length of each of these processes roughly equal to the length of the antenna. Antennae reddish, antennal funicle 3-segmented, antennal club oval, flattened, anterior side clearly marked by two procurved sutures, basal segment corneous, posterior side marked by two obscure rows of setae displaced apically. **Pronotum** 1.05–1.51 times longer than wide (1.05 in holotype), sub-quadrate, lateral, anterior and posterior margins slightly rounded from dorsal view, widest in the middle of its length, weekly declivous in front, summit not clearly marked, situated approximately in the middle, with a second summit just before the anterior margin, disk matt, of the same appearance as frons, shallowly, but very distinctly and rather densely punctured, punctuation becoming stronger towards pronotal base, granulated between punctures, surface strongly shagreened, impunctate median longitudinal area missing; vestiture of sparse, short semi-erected hair-like setae mixed with two times longer erected hair-like setae, the later becoming more dense and longer towards anterior margin. **Elytra** 1.31–1.91 times longer than wide (1.32 in holotype), 1.25–1.39 times longer than pronotum (1.26 in holotype), parallel on basal three-fourths, than converging to broadly rounded apical margin; base of elytrae rounded, not armed, sometimes a few blunt tubercles present as continuation of interstrial tubercles; scutellum visible, rather small, flush with elytra; elytral striae slightly impressed on elytral disk, deeply and densely punctured, punctures separated by less than the diameter of a puncture, interstriae narrower than striae, punctated and granulated, elytral suture very slightly elevated on the base of elytral declivity. Elytral declivity regularly rounded, of the same appearance as the elytral disk, except strial punctures and interstrial tubercles slightly larger, apical margin not armed, rounded. Elytral vestiture of semi-erect or erect, short, stout, sparse, uniseriate interstrial hair-like setae on the disk and declivity of elytra, their length is shorter than the distance between strial punctures, these setae becoming up to three times longer laterally and posteriorly. **Legs** dark brown. Procoxae subcontiguous, prosternal process narrow, sharply pointed, apically curved downward; mesocoxae broadly separated by distance of twice width of scapus, mesoventral process descending, broad and transversely truncated apically; metacoxae separated by similar distance as mesocoxae. Number of tibial socketed teeth on outer lateral margin varying between the specimens and between the right and left side. Protibiae with 4–5 socketed teeth, mesotibiae with 5–6 and metatibiae with 5 socketed teeth.

###### Female.

**Length** 1.33–1.89 mm (1.74 mm in allotype), 2.31–2.38 times longer than wide (2.35 in allotype). **Head** similar to male, but mandibulae not armed by tooth-like processes, frons convex, slightly flattened just above the epistoma, semi-matt, sparsely and finely punctate in the middle, punctuation getting very dense near epistoma on flattened area, nearly impunctate on vertex, whole surface shagreened; vestiture of gold, very sparse to missing short hair-like setae on vertex and becoming rather dense and long towards epistoma, anterior side of antennal club marked by two more strongly procurved sutures. **Pronotum** with lateral margins more narrowly converging anteriorly, punctuation of pronotum less distinct, tuberculation stronger.

##### Etymology.

Name of the new species is derived from Socotra – the island of its origin.

##### Biology.

Specimens were collected from wilting shrubs of Euphorbia arbuscula (Euphorbiaceae) in Hadiboh and Acacia pennivenia (Fabaceae) in Suq, additional specimens were obtained by rearing in the lab. Both host plants are endemic to Socotra. The newly described species is phloeophagous. The gallery system was not studied.

##### Distribution.

Yemen - Socotra, endemic.

#### Triotemnus kabateki

Knížek sp. n.

urn:lsid:zoobank.org:act:2BDB6B50-80BB-4767-B6A8-99C5F763B37A

Figs 11 14 

##### Type material.

Holotype male, pinned, with labels as follows: “Yemen, Soqotra Is., LAHAS/ (pass), 28.xi.2003, 12°13'46N; 54°05'26E, 69 m [GPS]/ leg. P. Kabátek, ex larva“; “YEMEN-SOQOTRA/ 2003/ Expedition: Jan Farkač, Pter Kabátek & David Král“; “Host plant: Euphorbia arbuscula”. Allotype female, pinned: the same data as the holotype. Paratypes: 30 males, 31 females: the same data as the holotype.

Holotype and Allotype deposited in the collection of National Museum in Prague, 57 paratypes in the author’s collection, 2 paratypes in Naturhistorisches Museum, Wien, 2 paratypes in Muséum d’Histoire Naturelle, Genève.

##### Diagnosis.

Triotemnus kabateki is morphologically distinct from all other species of the genus due to the unique male frons, slim body and small size in both sexes. The most closely related species is Triotemnus pilicornis Wood, 1992, which also has a conspicuous median spine on vertex. But the spine in Triotemnus pilicornis is dorso-ventrally flattened and the frons is not squeezed laterally. These species do not overlap in size (Triotemnus pilicornis is 1.5–2.2 mm long).

##### Description.

###### Male.

**Length** 0.96–1.22 mm (1.15 mm in holotype), 2.74–2.77 times longer than wide (2.67 in holotype), very slim and tiny species. Colour light to dark brown when fully coloured. **Head**. Frons strongly modified, deeply concave vertically, laterally squeezed, shining, smooth, impunctate and without any granules, on the median part of vertex with conspicuous, protuberant, large, pointed horn-like process; vertex semi-shining dorsally, shagreened and rather strongly punctuated near horn-like process, vestiture of frons of very sparse golden hair-like setae visible only from lateral view, horn-like structure on vertex hidden in long brush of hair-like setae directed straight forward and growing partly from the apex of the horn, but mainly on its dorso-lateral base. Eyes very small and triangle-shaped, placed on the latero-ventral margins of frons. Each mandible with rather long slim but strong sabre-shaped process directed anteriorly and curved in its last third inward, length of each of these processes roughly half of the length of the whole antenna. Epistoma and mandibles somehow protuberant, making the vertical excavation deeper from lateral view. Antennae yellowish, antennae funicle 3-segmented, antennal club oval, flattened, base on anterior side corneous, one transverse suture in the middle, apical half covered by dense setae, posterior side not marked by sutures. **Pronotum** 1.12–1.18 times longer than wide (1.12 in holotype), sub-cylindrical, widest in the middle of its length, lateral margins weekly rounded, frontal and posterior margins broadly rounded, pronotum moderately declivous on anterior third, summit approximately in the frontal third, disk shining, rather sparsely punctuated by course punctures, interspaces very finely shagreened, median longitudinal area impunctate; vestiture of sparse semi-erect hair-like setae, more fine than on elytra, becoming longer anteriorly. **Elytra** 1.59–1.63 times longer than wide (1.61 in holotype), 1.46–1.50 times longer than pronotum (1.50 in holotype), parallel on basal four fifths, than broadly rounded to apical margin; base of elytrae rounded, not armed, densely and deeply punctated; scutellum visible, very small, smooth and shining, flush with elytra; basal fifth of elytra very densely punctate and finely granulated, elytral striae not well defined, not impressed, whole elytra irregularly punctured, punctuation less deep than on pronotum, elytral suture very slightly elevated on the declivity. Elytral declivity regularly rounded, very slightly flattened in the continuation of second interstriae. Declivital disc of the same appearance as the elytral disk, but shagreened and semi-matt. Elytral vestiture of very sparse and minute, short, semi-erect, hair-like setae combined with slightly more abundant uniseriate, stout, hair-like erected setae that are approximately two times longer than the shorter setae, the longest becoming much longer postero-laterally, setae missing on the flattened second interstriae on the elytral declivity. **Legs** light brown. Procoxae separated by the width of scapus, prosternal process narrow, bluntly pointed; mesocoxae broadly separated by distance of twice the width of scapus, mesoventral process descending; metacoxae separated as mesocoxae. Number of tibial socketed teeth on outer lateral margin varying between the specimens. Protibiae with 4–5 socketed teeth, meso- and metatibiae with 5–6 socketed teeth.

###### Female.

**Length** 1.00–1.22 mm (1.11 mm in allotype), 2.71–3.34 times longer than wide (2.71 in allotype). **Head** similar to male, but mandibulae not armed by tooth-like processes, frons convex, slightly flattened above the epistoma, shining, very densely punctated on upper part, very minutely punctated on the flattened area, not well defined blunt and shining tubercle-like elevation on vertex; vestiture of golden, sparse, rather long, semi-erect hair-like setae.

##### Etymology.

The new species is named after my colleague Petr Kabátek, who collected the specimens. Among other, Petr Kabátek is the leading scientist in taxonomy and mainly biology of the beetle family Cerambycidae.

##### Biology.

Initial specimens were collected from wilting shrubs of Euphorbia arbuscula, endemic plant to Socotra, additional specimens were obtained by rearing in the lab. The newly described species is phloeophagous. The gallery system was not clearly discernable and mined in all directions close to the epidermis.

##### Distribution.

Yemen - Soqotra, endemic.

#### Triotemnus yemenensis

Knížek sp. n.

urn:lsid:zoobank.org:act:C8251172-B6D1-4287-83C6-06C25D800091

Fig. 12 

##### Type material.

Holotype female, pinned, with labels as follows: “SW YEMEN, Wadi Zabid E/ Zabid, 14°09’N; 43°31’E/ 325 m, 22. III. 2007/ leg. Petr Kabátek, ex larva“. Paratypes: 1 male, 3 females: the same data as the Holotype.

Holotype deposited in the collection of National Museum in Prague, Paratypes in the author’s collection.

##### Diagnosis.

Triotemnus yemenensis is morphologically readily distinct from all other species of the genus due to the small and stout body form, the very small mandibular teeth in the males, the very shining and sparsely, but strongly punctured pronotum in both sex. It may resemble Triotemnus subretusus Wollaston, but the latter species is slightly larger, the mandibular teeth are longer, the frons is not clearly punctated in males and is flattened in females, the pronotum is mostly very finely shagreened. Another morphologically similar species, Triotemnus scrofa Schedl, differs in the same characters and it also has a 3-segmented antennal funicle.

##### Description.

###### Female.

**Length** 1.26–1.30 mm (1.30 mm in Holotype), 2.50–2.78 times longer than wide (2.50 in Holotype). **Head**. Frons convex, shining, very finely shagreened, densely punctato-ganulate, granulation becoming more fine and dense towards epistoma, vestiture of golden, rather dense, long, erect, hair-like setae becoming more dense towards the epistomal margin. Eyes emarginate on anterior margin around the antennal insertion, rather large. Mandibulae not armed by tooth like processes. Antennae light brown, antennal funicle 4-segmented, antennal club round, rather strongly flattened, anterior side clearly marked by two weakly recurved sutures on anterior half, basal segment mainly corneous, posterior side without visible sutures. **Pronotum** 1.16 times longer than wide, dark brown to black, sub-oval, widest in posterior half, weekly declivous in front, summit not clearly marked, approximately in the frontal third, lateral margins converging anteriorly, frontal and posterior margins rounded, basal angles rounded, disk shining, rather sparsely punctated by coarse punctures except in a smooth median longitudinal area, interspaces finely shagreened, vestiture of sparse, long, semi-erect, very fine, hair-like setae, becoming slightly longer anteriorly. **Elytra** 1.46 times longer than wide, 1.34 times longer than pronotum, light brown, nearly cylindrical, widest just before declivity, broadly, nearly transversely rounded posteriorly; base of elytra rounded, not armed; scutellum visible, very small, blackish, flush with elytra; elytral striae regularly, finely and sparsely punctate, not impressed, interspaces slightly larger then the diameter of the strial punctures, interstriae very sparsely and less deeply punctated, smooth and finely shagreened; elytral declivity regularly rounded at suture, otherwise flattened in the space between suture and third striae up to the weakly defined lateral edges which are slightly elevated, elytral suture very slightly elevated, declivital disc smooth, shining, finely shagreened, microscopically punctate in continuation of the elytral striae. Elytral vestiture of uniseriate rows of two types, sparse and very long (approximately 1.5 times longer than distance between strial punctures) hair-like erect setae and semi-erect shorter (two thirds of the longer setae) hair-like setae, long setae becoming much longer posteriorly on the declivity. **Legs** light brown. Procoxae very narrowly separated, prosternal process short and sharply pointed; mesocoxae separated by slightly more than double width of scapus, mesoventral process broad and broadly rounded apically; metacoxae separated similarly to mesocoxae. Number of tibial socketed teeth on outer lateral margin varying between the specimens. Protibiae with 4–5 socketed teeth, mostly 4, meso- and metatibiae with 5–6 socketed teeth, mostly 5.

###### Male.

**Length** 1.17 mm, 2.54 times longer than wide. **Head** similar to female, but frons very shallowly concave up to the level just above upper level of the eyes, concavity very finely and sparsely punctate, shining, very finely shagreened, excavation separated from vertex by a rounded and not well defined costa, marked on vertex in the middle by short transverse keel, vertex semi-shining, deeply and rather densely punctuate; vestiture of frons of very short and fine semi-erect golden, hair-like setae, these apparently longer near epistoma and on the upper border of the frontal excavation. Each mandible with a very short, strong, wide-based, tooth-like process directed upward.

##### Etymology.

Name of the new species is derived from Yemen – the country of its origin.

##### Biology.

Specimens were collected on wilting shrubs of Euphorbia sp. The newly described species is phloeophagous. The gallery system was not studied.

##### Distribution.

Yemen, perhaps endemic.

## Supplementary Material

XML Treatment for Triotemnus
